# The response of anti-VEGF therapy and tamoxifen withdrawal of tamoxifen-induced cystoid macular edema in the same patient

**DOI:** 10.1186/s12886-021-01953-z

**Published:** 2021-05-07

**Authors:** Chuanyu Li, Jun Xiao, He Zou, Bo Yang, Lifu Luo

**Affiliations:** Second affiliated Hospital of Jilin University, Changchun, China

**Keywords:** Tamoxifen, Rentinopathy, Cystoid macular edema, Anti-VEGF therapy, Drug withdrawal

## Abstract

**Background:**

Numerous cases with ocular toxicity secondary to tamoxifen have been reported, and became more apparent with keratopathy, cataract, optic neuritis, macular holes, crystalline retinopathy with or without cystoid macular edema (CME). Withdrawing tamoxifen with the approval of the oncologist is the major treatment for cases with tamoxifen-induced retinopathy.

**Case presentation:**

We herein reported a patient with a two-year history of painless and reduced visual acuity in both eyes who received tamoxifen therapy for 6 years. Tamoxifen-induced rentinopathy with CME showed significant development even though the patient has already discontinued tamoxifen treatment for 6 months. Anatomic improvements after intravitreal ranibizumab injection in both eyes were significant but were temporary. Surprisingly, CME in both eyes has been resolved spontaneously after 10 months in the penultimate visit without any therapy.

**Conclusion:**

Intravitreal ranibizumab injection temporarily improved the anatomy of the eyes in a case with tamoxifen-induced CME, and only tamoxifen withdrawal can bring a sustained effect.

## Background

Tamoxifen is a nonsteroidal antiestrogen that is primarily approved for reducing the risk of breast cancer recurrence in high-risk female patients at low doses (20 mg daily) [1]. Besides systemic side effects like menopausal symptoms, thromboembolic effects, and ocular symptoms, including keratopathy, cataract, optic neuritis, macular holes, crystalline retinopathy with or without cystoid macular edema (CME), and pseudocystic foveal cavitation have been mainly reported in cases who have taken high cumulative doses [1–3]. Recent reports have demonstrated that tamoxifen-induced CME can be relieved by treatment with anti-vascular endothelial growth factor (anti-VEGF) or intravitreal triamcinolone acetonide [4]. We herein reported a case of severe tamoxifen-induced CME, and observed the response of ranibizumab use with long term follow-up.

## Case presentation

A 52-years-old Han Chinese woman with a two-years history of painless and reduced visual acuity in both eyes without any metamorphopsia and color anomalopia was examined at the Department of Ophthalmology, Second Affiliated Hospital of Jilin University in May 2018. She has been diagnosed with left-side breast cancer, and given tamoxifen 80 mg per day for 6 years since 2011. A modified radical mastectomy was performed and postoperative chemotherapy was given for 6 times in 2015. Pathology results revealed an invasive ductal carcinoma with negative left axillary lymph nodes. At the time of presentation, the patient was 6 years postmenopausal and discontinued tamoxifen treatment for 6 months and the total dose of tamoxifen at that time was greater than 170 g. The developmental history as well as the family history remained unremarkable, and no previous history of diabetes and hypertension was present.

**Fig. 1:**
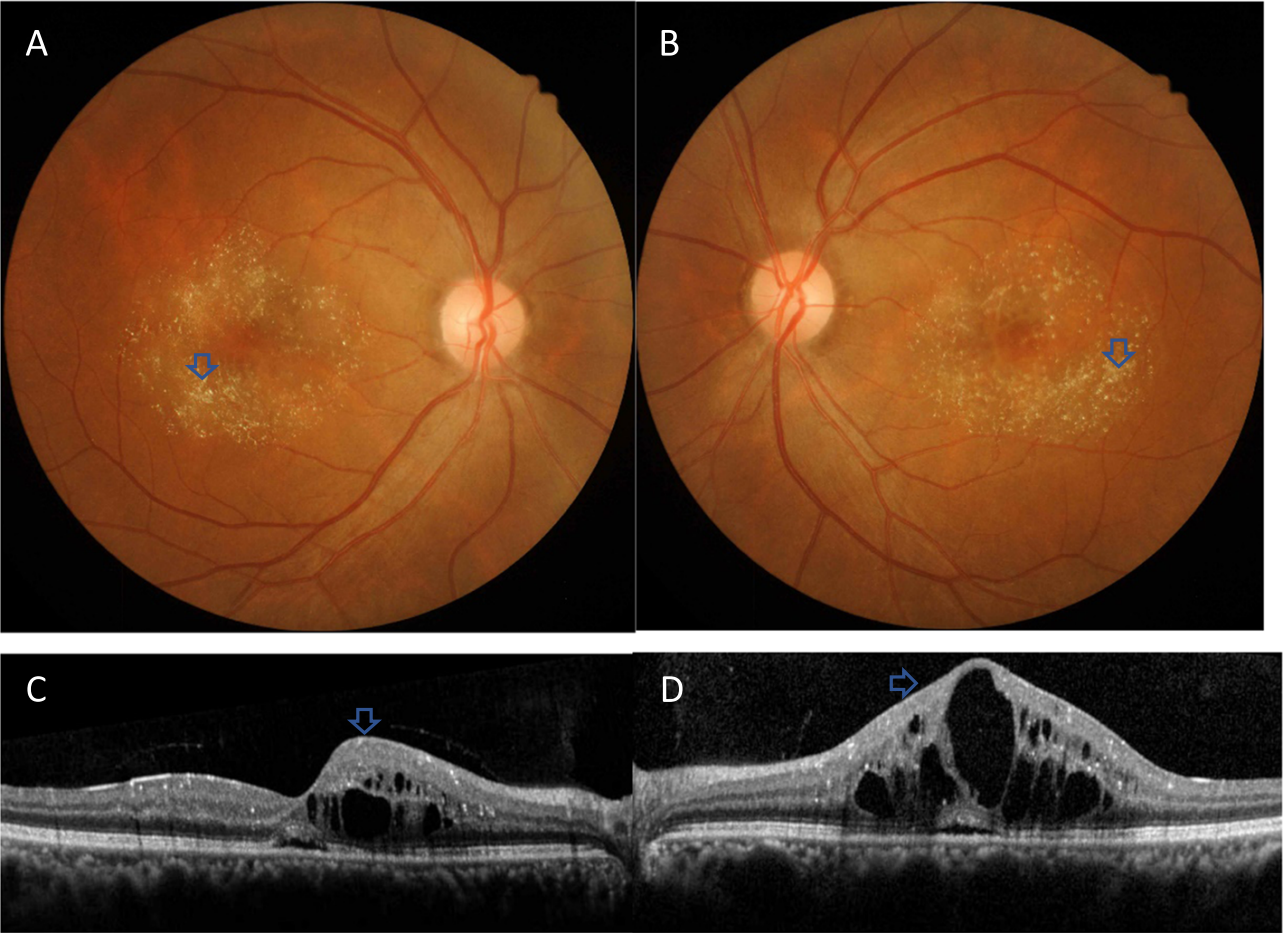
CFP and OCT taken in May 2018. Fundus examination revealed yellow-white refractive deposits in the macular and paramacular areas in the right eye (**a**) and the left eye (**b**). OCT showed CME and refractile deposits in superficial retinal layer, while mild subretinal fluid can also be found in both eyes (**c**, **d**)

**Fig. 2:**
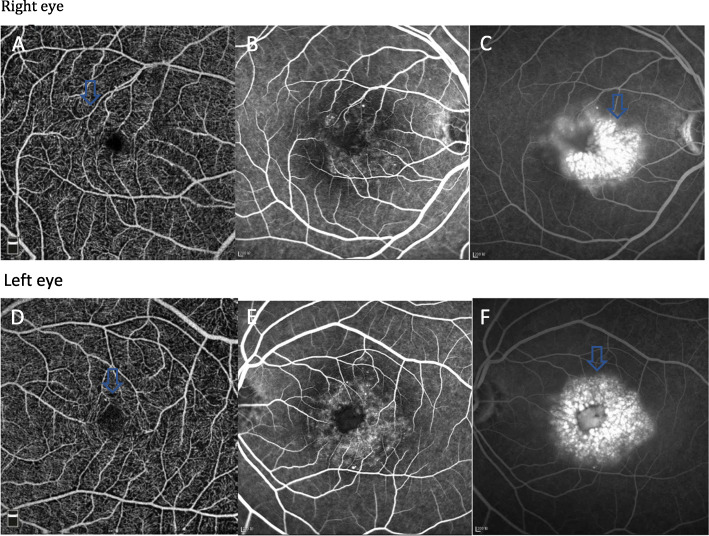
OCTA and FFA taken in May 2018. OCTA (**a**, **d**) in the superficial layer and FFA in the arteriovenous phase (**b**, **e**) of both eyes showed telangiectasia. FFA revealed a typical petalloid-like pattern of hyperfluorescence and dye leakage in the macula during the late phase (**c**, **f**)

On examination, her best corrected visual acuity (BCVA) was 20/40 in the right eye and 20/80 in the left eye. The anterior segment examination and intraocular pressure in both eyes were normal. Fundus examination revealed yellow-white refractive deposits in the macular and paramacular areas (Fig. 1a and b). The spatial domain optical coherence tomography (SD-OCT) imaging showed CME, refractile deposits in both eyes, and macular white crystalline deposits in the superficial retinal layers (Fig. 1c and d). Optical coherence tomography angiography (OCTA), (Fig. 2a and d) of both eyes showed telangiectasia. Fundus fluorescein angiography (FFA) in the arteriovenous phase (Fig. 2 b and e) showed telangiectasia, while a typical, petalloid-like pattern of hyperfluorescence was observed in the late phase (Fig. 2 c and f). Similar changes were seen in both eyes.

Considering the overall clinical picture and the recent use of tamoxifen, the patient was considered to be finally diagnosed with tamoxifen-induced rentinopathy.

**Fig. 3:**
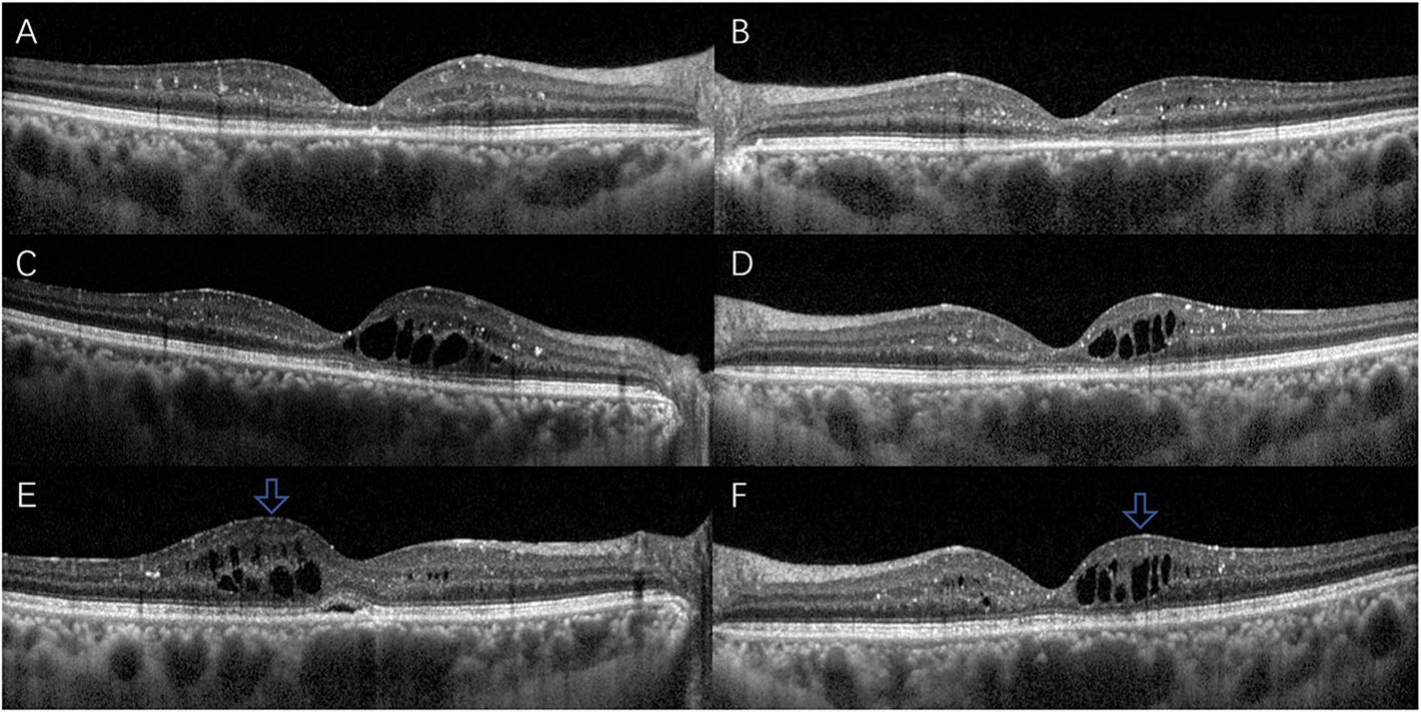
Optical coherence tomography scans 1 month after the initial injection revealed no edema in the right eye (**a**) and left eye (**b**). **c** and **d** showed first recurrence during the therapy process. Optical coherence tomography scans revealed mild CME in right eye (**e**) and left eye (**f**) 10 months after the initial presentation

**Fig. 4:**
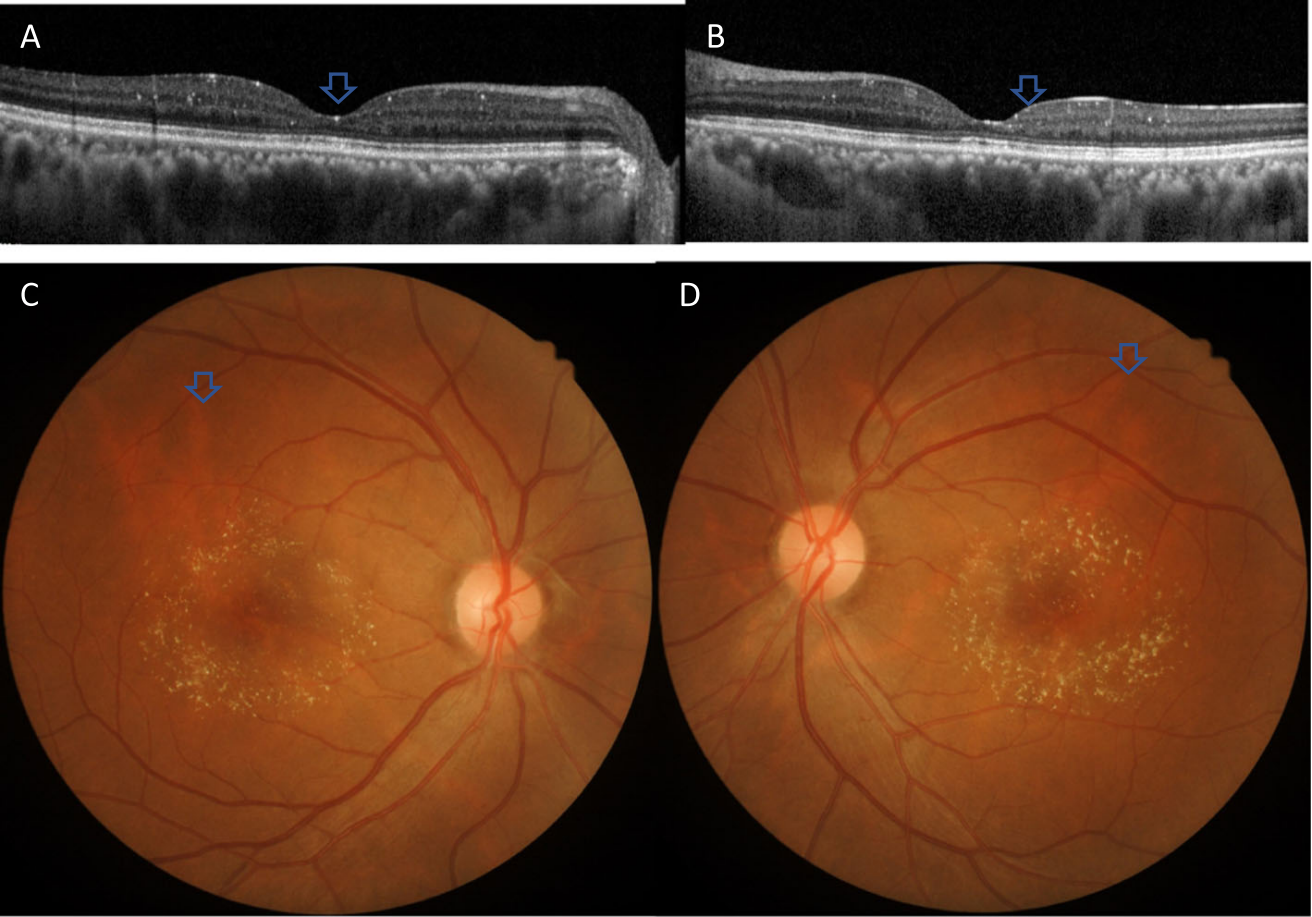
CFP and OCT taken in April 2020. Optical coherence tomography scans 23 months after the initial presentation revealed no edema in the right eye (**a**) and left eye (**b**). The yellow-white refractory deposits showed slight reduction when compared with the first visit (**c**, **d**)

Both eyes were subsequently treated with intravitreal ranibizumab 0.5 mg injection. The patient received first injection to the left eye in May 2018 followed by the right one a month later. The patient was followed up for 23 months including 1 week after the first injection to the left eye. The patient received reviews every month for the first 6 months after the first injection. Color fundus photography (CFP) and SD-OCT were performed at each visit. Also no significant CME was observed 1 month after the injection to both eyes (Fig. 3 a and b). Her BCVA was 20/40 in the right eye and 20/50 in the left eye. However, the best condition in the right eye was maintained only for less than 2 months (Fig. 3 c). Therefore, the patient received second intravitreal ranibizumab injection in the right eye and the improvements lasted for only 3 months. Compared with the right eye, normal retinal anatomical structures were observed in the left eye, which lasted for nearly 3 months following the injection. CME showed recurrence 3 months after the first injection (Fig. 3 d). Considering the unsatisfied visual acuity (VA) gain and the economic burden, the patient refused anti-VEGF therapy but continued follow up with CFP and OCT. SD-OCT still showed mild CME in both eyes 10 months after the first presentation in her penultimate visit, i.e., on March 2019 (Fig. 3 e and f). However, CME in both eyes was completely solved (Fig. 4 a and b) at the next and last follow-up visit in April 2020. The yellow-white refractory deposits showed slight reduction when compared to the first visit (Fig. 4 c and d) and BCVA was 20/40 in the right eye and 20/63 in the left eye.

## Discussion and conclusions

Ocular toxicity secondary to the use of high-dose (240–320 mg/day) tamoxifen was first described in 1978 by Kaiser-Kupfer and Lippman. Later, it was found that smaller doses (20–40 mg/day) of tamoxifen showed a foveal cyst with disruption of the photoreceptor line, while a high-dose therapy resulted in typical CME [2, 5, 6]. The yellow-white refractive deposits are typical of tamoxifen-induced rentinopathy as well [2]. Tamoxifen-related CME occurred 4 years after the treatment, and this change was presented for over 17 to 27 months after tamoxifen treatment [2]. Pathogenesis of tamoxifen toxicity still remained to be unknown. In vitro, tamoxifen acted as an antagonist to glutamate transporters in retinal pigment epithelial cells. The increased glutamate in the outer retina resulted in axonal degeneration and Müller cell impairment [2]. Being cationic and amphophilic, tamoxifen had a hydrophobic moiety and a positively charged hydrophilic side chain on the same molecule, resulting in in vivo accumulation of drug-polar lipid complexes in the lysosomes [7]. The deposits might consist of drug-polar lipid complexes, and showed no improvement even after long-term withdrawal of tamoxifen but showed slight reduction when 23 months after initial presentation (Fig. 4 c and d) of our case.

Tamoxifen-related macular edema is not mediated by VEGF. Tamoxifen might actually decrease the serum VEGF as reported previously [4]. The pathogenesis of macular edema presumably involves vascular endothelial damage and increased vascular permeability [4]. One case showed similar CME as reported in our case who received 7 triamcinolone injections in each eye for over 20 months but still required to continue intermittent injections [4]. As see in our case, the tamoxifen-related macular edema showed recurrence after intravitreal ranibizumab injection and then was resolved spontaneously without undergoing any specific therapy. From this, we concluded that temporary anatomic improvement was correlated with intravitreal ranibizumab injection. The anatomic development during follow-up in our case can be explained in two ways, one is related to anti-VEGF therapy while the other is related to tamoxifen withdrawal. In the first method, the development is temporary and is mainly because of decreased vascular permeability, while the other one remains stable. Dr. Ehsan reported that the patient with tamoxifen included CME who have no improvement 7 months after cessation of tamoxifen finally benefitting from intravitreal bevacizumab injection [8]. In contrast, in our case anti-VEGF therapy remains to be helpful but is temporary and tamoxifen withdrawal can bring a sustained effect.

What cannot be ignored is that a similar etiology between tamoxifen-induced rentinopathy and MacTel2 of decreased serum estrogen concentration and functional damage of Müller cells caused similar symptoms [9, 10]. Both of these can present CME and yellow-white deposits. In our case, the medical history can be helpful in making differential diagnoses. In addition, the CME of MacTel2 cannot be resolved spontaneously unlike the tamoxifen-related macular edema with drug withdrawal. The basic intact retinal tissue after long-term visit also supported this diagnosis.

As we can see, intravitreal ranibizumab injection can be used to maintain normal retinal anatomical structures in our patient with tamoxifen-related macular edema. However, withdrawing tamoxifen with the approval of the oncologist is considered as the major treatment for tamoxifen-induced rentinopathy at present.

## Data Availability

All data generated or analysed during this study are included in this published article and its supplementary information files.

## Abbreviations

VEGF: Vascular endothelial growth factor
CME: Cystoid macular edema
BCVA: Best corrected visual acuity
SD-OCT: Spatial domain optical coherence tomography
OCTA: Optical coherence tomography angiography
FFA: Fundus fluorescein angiography
CFP: Color fundus photography
VA: Visual acuity

## References

[1] Chung SE, Kim SW, Chung HW (2010). Estrogen antagonist and development of macular hole. Korean J Ophthalmol.

[2] Makri OE, Georgalas I, Georgakopoulos CD (2013). Drug-Induced Macular Edema. Drugs.

[3] Wang L, Miao H, Li X (2015). Tamoxifen retinopathy: a case presentation. SpringerPlus.

[4] Jeng KW, Wheatley HM (2015). Intravitreal triamcinoloneacetonide treatment oftamoxifen maculopathy withassociated cystoid macular edema. Retin Cases Brief Rep.

[5] Bommireddy T, Zia Iqbal Carrim B. To stop or not? Tamoxifen therapy for secondaryprevention of breast cancer in a patient withocular toxicity. BMJ Case Rep. 2016. 10.1136/bcr-2015-213431. 10.1136/bcr-2015-213431 PMC4716368 26759403

[6] Rijal RK, Nakhwa C, Sindal MD (2014). Crystalline deposits in the macula – tamoxifen maculopathy ormacular telangiectasia?. Nepal J Ophthalmol.

[7] McKeown CA, Swartz M, Blom J (1981). Tamoxifen retinopathy. Br J Ophthalmol.

[8] RAHIMY E, SARRAF D (2012). Bevacizumab therapy for tamoxifen-induced crystalline retinopathy and severe cystoid macular edema. Arch Ophthalmol.

[9] Behrens A, Sallam A, Pemberton J (2018). Tamoxifen Use in a patient with idiopathic macular telangiectasia type 2. Case Rep Ophthalmol.

[10] Moussa K, Kim J, Miller JB (2019). Retinal exudatesand hemorrhages in a survivor of breast cancer. JAMA Ophthalmol.

